# Investigation of Machine and Deep Learning Techniques to Detect HPV Status

**DOI:** 10.3390/jpm14070737

**Published:** 2024-07-10

**Authors:** Efstathia Petrou, Konstantinos Chatzipapas, Panagiotis Papadimitroulas, Gustavo Andrade-Miranda, Paraskevi F. Katsakiori, Nikolaos D. Papathanasiou, Dimitris Visvikis, George C. Kagadis

**Affiliations:** 1 3dmi Research Group, Department of Medical Physics, School of Medicine, University of Patras, 26504 Rion, Greecekagadis@upatras.gr (G.C.K.); 2 Laboratoire de Traitement de l’Information Médicale (LaTIM), UMR 1101, INSERM, University of Brest, 29200 Brest, France

**Keywords:** cancer, HPV status, deep learning, artificial intelligence, ensemble models

## Abstract

Background: This study investigated alternative, non-invasive methods for human papillomavirus (HPV) detection in head and neck cancers (HNCs). We compared two approaches: analyzing computed tomography (CT) scans with a Deep Learning (DL) model and using radiomic features extracted from CT images with machine learning (ML) models. Methods: Fifty patients with histologically confirmed HNC were included. We first trained a modified ResNet-18 DL model on CT data to predict HPV status. Next, radiomic features were extracted from manually segmented regions of interest near the oropharynx and used to train four ML models (K-Nearest Neighbors, logistic regression, decision tree, random forest) for the same purpose. Results: The CT-based model achieved the highest accuracy (90%) in classifying HPV status. Among the ML models, K-Nearest Neighbors performed best (80% accuracy). Weighted Ensemble methods combining the CT-based model with each ML model resulted in moderate accuracy improvements (70–90%). Conclusions: Our findings suggest that CT scans analyzed by DL models hold promise for non-invasive HPV detection in HNC. Radiomic features, while less accurate in this study, offer a complementary approach. Future research should explore larger datasets and investigate the potential of combining DL and radiomic techniques.

## 1. Introduction

Oropharyngeal squamous cell carcinoma (OPSCC) is one of the most frequent and fastest-growing head and neck cancers (HNCs). Infection with high-risk human papillomavirus (HPV), particularly type 16, has been recognized as a risk factor in this type of cancer [1], as well as tobacco and alcohol consumption [2,3]. In addition, many studies suggest that although both HPV-positive and HPV-negative OPSCCs refer to the same anatomical location, they demonstrate several biological and clinical differences [4]. Consequently, the HPV status of the patient affects and determines not only the diagnosis but the therapy and the follow-up plan as well.

Regarding the conventional treatment of OPSCC, surgery, radiotherapy, chemotherapy, and immunotherapy are the main options and are performed individually or in combination, depending on the TNM (Tumor Nodes Metastasis) class and the primary site. When it comes to the detection of HPV infection, the in situ hybridization (ISH) of viral DNA, HPV DNA, or RNA PCR and immunohistochemical (IHC) investigation for p16 overexpression are the most common methods [5]. However, in cases of the latter, the testing requires a biopsy sample, which makes the procedure invasive, and thus, patients can be exposed to surgery-related complications and risks [4]. As a result, there is a great need for non-invasive, yet accurate methods for the detection of the HPV infection.

Due to tumor heterogeneity, which is a very common prognostic factor in HNC cases, a personalized treatment plan is deemed necessary to improve the survival rate and minimize possible side effects [6,7]. In recent years, radiomics has emerged as a potential alternative tool and has attracted a lot of attention [8]. It is a field of computational medicine that allows the automated high-throughput extraction of excessive amounts of quantitative features from two-dimensional or three-dimensional medical images, which can be combined with different pathology and molecular biomarkers, to evaluate the state of a tumor with great accuracy and create a personalized diagnosis and treatment plan for patients [9,10,11]. For the analysis of such data, artificial intelligence (AI) techniques are usually employed such as machine learning (ML) algorithms.

Over the past decade, increases in computational power and memory have led AI to significant strides in transforming the landscape of healthcare and medicine, using technologies that empower machines to interpret, analyze, and learn from complex medical data, leading to more accurate and efficient decision-making processes [12,13]. These technologies include ML, neural networks (NNs), and Deep Learning (DL).

The aim of the present study was to develop alternative and non-invasive approaches that analyze computed tomography (CT) data and radiomic features to classify the HPV status of patients with HNC. Usually, such classification is performed using and combining microscopic diagnosis methods results (Immunohistochemistry—IHC, in situ hybridization—ISH, Polymerase Chain Reaction—PCR), which are time consuming and add stress to the diagnosis procedure. The proposed approach aims to exploit additional imaging data, such as CT scans from patients, to support clinicians toward HPV detection. Initially, CT data were used to train a modified Residual Network with 18 layers (ResNet-18) [14] to predict the HPV status, and secondly, 15 radiomic features were extracted from a ROI near the oropharynx and were utilized to train four different ML classifiers, aiming to achieve the same result. Various metrics were implemented to evaluate the performance of each model. Furthermore, the Weighted Ensemble method was employed on the predictions to combine the CT-based model with one of the radiomics-based models, to create improved predictions, and to calculate corresponding metrics. Finally, the predictive value of each technique was investigated to highlight the main challenges and to reach conclusions concerning the possibility of including such models in clinical routine as a supplementary tool for clinicians.

## 2. Materials and Methods

**Figure 1:**
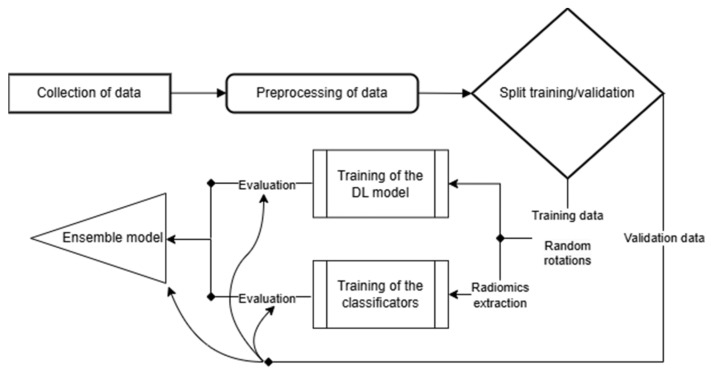
The flowchart of the procedure followed in the proposed approach.

For completeness, the flowchart of the complete procedure is included in Figure 1.

### 2.1. Patient Dataset

From the HECKTOR database [15], fifty (50) CT data that referred to patients with histopathologically confirmed oropharyngeal HNC who underwent treatment planning involving radiation therapy and/or chemotherapy were included in the study. The dataset includes FDG-PET and low-dose non-contrast-enhanced CT images that were acquired through combined PET/CT scanners, focusing on the HNC region, as well as the gross tumor volume (GTV) mask. In our study, we only used the CT data. In addition, as shown in Table 1, there is also patient-specific information including positioning details, age, gender, primary tumor site, TNM stage, overall stage, HPV status, and the possibility of chemotherapy as an additional treatment after radiation therapy. In addition, the data are sourced from three different clinical centers to ensure multi-centric variability on the used dataset, being able to not overparameterize the proposed models on a specific clinical system. More specifically, those centers are as follows:

1. Hôpital Général Juif (CHGJ) in Montréal, QC, Canada (*n* = 12);
2. Centre Hospitalier Universitaire de Sherbooke (CHUS), Sherbrooke, Canada (*n* = 19);
3. Centre Hospitalier Universitaire de Poitiers (CHUP), Poitiers, France (*n* = 19).

In Table 1, we analytically present the dataset used during the training and validation process of our models. The choice of data, as well as the pre-processing, aimed to create a heterogeneous and harmonized dataset. This dataset would not be limited to specific types of data, but a wider independent selection. That being said, the dataset would be independent of the hospital, or the system used, as well as independent of the status of the patient. Hybrid PET/CT scanners, Discovery ST by GE Healthcare (Chicago, Illinois, United States) for the CHGJ, GeminiGXL 16 by Philips (Amsterdam, Netherlands) for the CHUS, and Biograph mCT 40 ToF by Siemens (Erlangen, Germany) for the CHUP, were used for image acquisition. Data augmentation including a larger population of patients would improve the performance of the proposed models.

### 2.2. Radiomic Features Extraction and Selection

**Figure 2:**
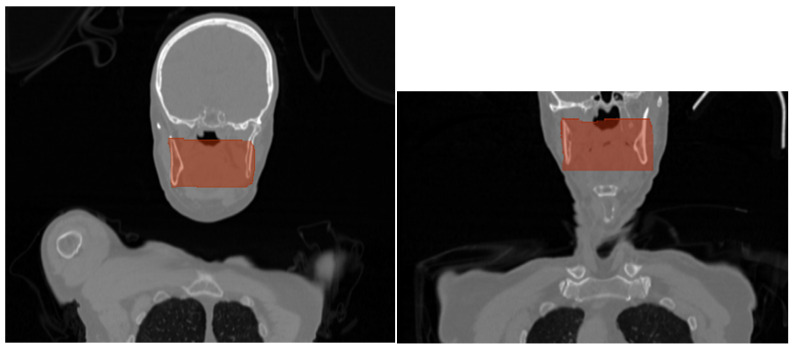
Representative slices, with the manual segmentation of the ROI.

**Figure 3:**
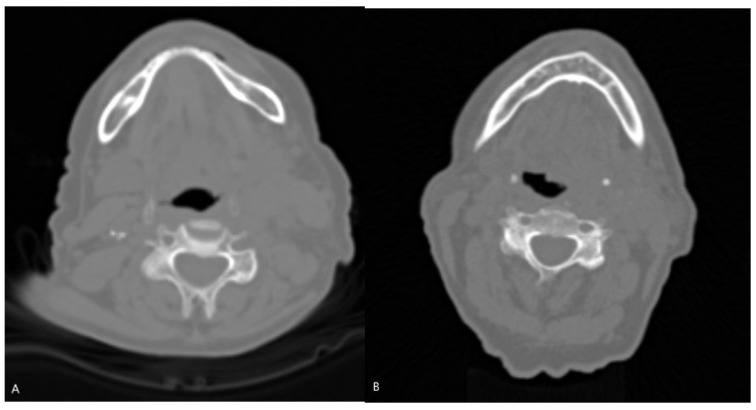
Representative slices of patients. (**A**) An HPV-negative 59-year-old woman. (**B**) An HPV-positive 74-year-old man.

CT data were organized in an NIfTI format for convenient data management. 3D Slicer [16], which is a free and open-source software platform used for medical image processing and visualization, was used for radiomics extraction. 3D Slicer’s segmentation tools, and more specifically, the segment editor, was used to manually delineate regions of interest (ROIs) that needed to be analyzed. In this case, the ROI was the wider area of the oropharynx, and the segmentation was performed on ten slices and for every patient in the dataset. Those ten slices were chosen from the area of HNC, based on the malignancy, as well as on the probable position of lymph nodes. The area was not segmented in detail, but in a wider cubic region of interest (ROI), aiming to develop a method that will save time from clinicians using fixed ROIs (Figure 2). In Figure 3, CT slices from an HPV-positive and an HPV-negative patient are presented. As shown in Figure 2, the manual segmentation of the ROI was performed in one of the ten slices of these patients.

Furthermore, a radiomics extension of 3D Slicer named SlicerRadiomics, which encapsulates a Python package (v3.7.0) commonly used for radiomics analysis, called PyRadiomics [17], was utilized. Feature extraction within SlicerRadiomics involves utilizing algorithms and techniques to analyze medical data from different medical images, like CT and MRI scans. These methods identify and quantify characteristics like texture, shape, and intensity in the images, employing filters, segmentation, and statistical analysis. The extracted features can provide valuable information for tasks such as disease characterization, classification, treatment response evaluation, and outcome prediction.

Top of Form extension allows the bin width to be set to 25 and radiomic features to be extracted, such as shaped-based, first-order, and second-order features. Based on the literature [11,18,19,20], regarding HNC and HPV status classification, 15 features that are considered relevant to such studies were used. As these features are already considered relevant to such classification, this study did not apply additional elimination procedures. Only features that were not considered important by already-published studies investigating the HPV status and that were produced by PyRadiomics were eliminated. More specifically, the following features were used:

- First-order features (Entropy, Kurtosis, Mean, Skewness, Uniformity)
- Second-order features related to the Gray Level Co-occurrence Matrix (Contrast, Correlation, Inverse Variance, Joint Energy), the Gray Level Size Zone Matrix (Small Area Emphasis, Large Area Low Gray Level Emphasis), and the Neighboring Gray Tone Difference Matrix (Busyness, Complexity, Coarseness, Contrast).

### 2.3. Pre-processing and Preparation of Data

In this study, the pre-processing of the data and thus the training and the evaluation of models were separated into two phases. The first refers to the use of the CT data for the training of a DL model, originated by ResNet-18, which is an image classification convolutional neural network that is 18 layers deep, pre-trained on ImageNet dataset [21]. The second phase refers to the use of radiomic features for the training of several machine learning models. The training and evaluation parts will be explained in the following chapters.

Before using CT data as an input for training the model, several preprocessing steps are needed to ensure that they are suitable to be used with the model. Initially, data were normalized to a range between 0 and 1, as this is necessary for machine and DL algorithms to optimally perform when dealing with inputs that include data of different ranges. More specifically, this kind of model can be sensitive to the scale of the input data; thus, by normalizing the pixel values, models become less sensitive to variations in input intensity, and thus, the generalization to unseen data highly improves. In addition to the normalization, each slice of the CT data was resized to 224 × 224, as most pre-trained models expect a fixed input size. Using such a size also helps the algorithm to be trained, as using large images (e.g., 1024 × 1024) results in too much information to be handled by the DL architectures. To use less space when storing the images, we converted them to float 16. In addition, it is important to use standard input sizes instead of dealing with varying dimensions to avoid the potential loss of spatial information. Next, due to the small amount of data, each 3D image was considered as a stack of 2D slices, resulting in a larger amount of data used for the training of our model. However, it is important to mention that this process is insufficient in capturing 3D contexts. Nevertheless, it is important to mention that for the radiomics models, as a cubic ROI was used, they considered volume heterogeneities.

For the radiomics-based models, the radiomic features were defined as variable X (i.e., list) and the HPV status label (0 for HPV− or 1 for HPV+) as the y variable. The one-hot encoding method was utilized to encode the independent variable, while label encoding was used to encode variable y. In this way, it was ensured that the data were in a compatible format with the algorithms, as most of them rely on numerical representations of features for interpretability and learning. Moreover, feature scaling was performed to ensure that all features made an equal contribution to the learning process, preventing certain features from dominating others and making the optimization procedure more stable.

### 2.4. Modeling and Training

As mentioned, two different types of models were trained, considering two different types of data as input in each case. All the models and the data that were used in the context of this study can found in GitHub [22]. As a first step, a pre-trained DL model, called ResNet-18, was trained, using the CT data of patients as inputs. The next step was to train 4 pre-trained ML models (KNN, LR, DT, RF) using the 15 radiomic features that we extracted from the CT images as input.

For the training of the CT-based model, the transfer learning technique was applied on ResNet-18. Transfer learning is a method in ML where a model initially trained for one task is adjusted and refined to suit a different yet interconnected task. This technique enables the utilization of knowledge gained from pre-training on a large dataset for a different but related task. This proves beneficial when the target task, such as the aim of this study, lacks sufficient labeled data. In addition, pre-trained models, having learned useful features from diverse and multiple data, accelerate the convergence of the model, something that is advantageous in cases where computational resources or time are limited. Transfer learning often results in more robust models, as features learned from diverse data contribute to a better understanding of underlying patterns, making the model less sensitive to variations.

ResNet-18 consists of an input layer that takes an input image, typically in a square format, with fixed dimensions (i.e., 224 × 224). Then, there are convolution layers, which include convolutional operations, batch normalization, and rectified linear unit (ReLU) activation functions to extract features from the input image. Some of them employ down-sampling, achieved through increased stride or pooling operations, to reduce spatial dimensions. In addition to convolutional layers, there are residual blocks, each one comprising two convolutional layers with batch normalization and ReLU activation. A skip connection is included to allow a direct flow of gradients during backpropagation. The final layer (output layer) involves a fully connected layer with a SoftMax activation function, generating class probabilities in classification tasks. Sometimes, instead of using the conventional fully connected layers for classification, ResNet-18 utilizes global average pooling (GAP), a method that involves condensing the spatial dimensions of each channel to a single value, providing a more compact representation.

In this study’s implementation, the input channels were modified from 3 to 1, as CT data are not in RGB format, with a kernel size of (7, 7), a stride, and padding equal to (2, 2) and (3, 3), respectively. Additionally, 1 output dimension was implemented instead of 1000, as this case focuses on a binary classification. For the loss function and the optimizer, the Binary Cross Entropy with Logits Loss function and the Adam Optimizer were utilized, implementing a learning rate equal to 0.0001.

**Figure 4:**
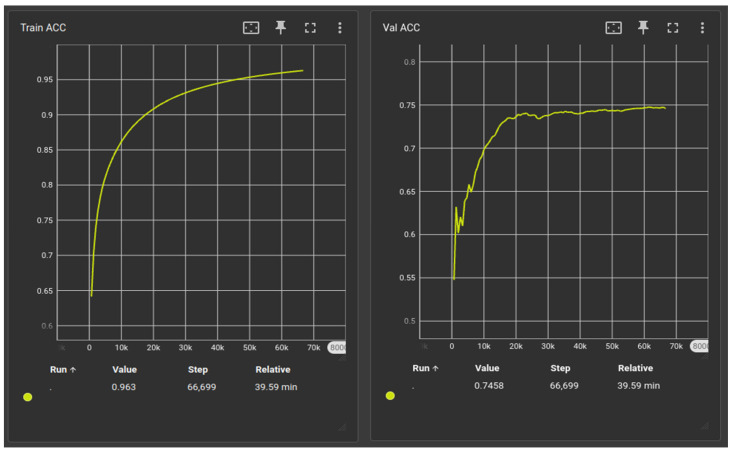
Training and validation accuracy during the training of the CT-based model.

To train the model, the dataset of the 50 patients was split into two smaller datasets: the training set, including 40 patients, and the validation set, including 10 patients. Given that the chosen data by the HECKTOR database were limited, to improve the model’s generality, they were resampled as 2D radiology data. In addition to the utilization of large and additional data generated, resampling also improves the consumption of time, cost, and labor. Hence, the CT images were represented as 2D slices, and the total number of 2D slices that were used for the training was 7195, ensuring adequate generality and robust results for testing on unseen data. Moreover, the training set was used for the training of the model, while the validation set was only used for its evaluation. The training was performed in batches of 16 slices. During the process, the training and validation accuracy were monitored to assess the point where the values began to become stable (Figure 4) and avoid overfitting. To avoid overfitting, data from various clinical centers were included, and the model was trained in different random amounts of epochs, before deciding the proper amount of training needed. A total of 100 epochs seemed to be the proper spot where the model performed very well, with high training and validation accuracy, as shown in Figure 4.

As radiomics-based models, K-Nearest Neighbors, logistic regression, decision tree, and random forest were implemented. After preprocessing with 3D Slicer and the PyRadiomics tool, radiomics features were used as input in each model to start the training. The dataset was split following the same procedure as in the CT-based model, with 40 patients as the training set and 10 patients as the validation set. More specifically, for the logistic regression classifier, the “random state” parameter was set to 0 to ensure reproducibility. For the K-Nearest Neighbors, the number of neighbors was set to 5, meaning that the algorithm would consider the labels of the five nearest neighbors when making predictions. Also, “Minkowski” was utilized for calculating the distance between points, and p was set to 2, corresponding to the Euclidean distance. Regarding the decision tree model, the criterion that was used for measuring the quality of a split was “entropy”, which means that the algorithm would use the information gained based on entropy to decide the best splits. The random state was set to 0 to make sure that the results would be reproducible. For the random forest classifier, the same parameters as in the decision tree were implemented, while the number of estimators was set to 10, meaning that the ensemble would consist of 10 decision trees. After training, the prediction for each case to be 1 (HPV positive) was utilized for the performance metrics.

### 2.5. Ensemble Methods

Ensemble methods in machine learning involve the combination of predictions from two or more models in some manner (e.g., averaging or voting) to enhance their overall performance and generalization. There are different types of ensemble methods, but in the context of this study, the Weighted Ensemble method was implemented. In this method, each model is assigned a weight that reflects its significance, and thus contributes to the final prediction. Higher weights are typically assigned to models that exhibit better performance or are considered more reliable.

A simple ensemble approach involves using N predictors denoted as C_1_, …, C_N_ and applying a function f to combine their predictions. For each instance x that needs prediction, the ensemble generates predictions using the function f(C_1_(x), …, C_N_(x)), where Ci(x) represents the prediction of predictor C_i_ for instance x. In the context of classification, the function f can be a voting mechanism, which essentially selects the most frequently predicted class among the C_i_(x). In weighted voting, each predictor C_i_ is assigned a weight w_i_ for its vote. In a two-class situation with classes, the weighted voting process can be expressed as follows:

(1) $$prediction\left(x\right)=\left\{\begin{array}{l} 1\;if\;\sum_{i}w_{i}C_{i}\left(x\right)>\theta \\ 0\;\mathrm{otherwise} \end{array}\right.,$$

where *θ* is a suitable threshold.

In this study, the Weighted Ensemble method was applied four times in total, on two models each time: the CT-based model with each machine learning model, separately. Firstly, their weights were defined, based on their performance metrics. More specifically, for the models that had results formed in possibilities, 0.6 was assigned as weight to the CT-based and 0.4 to the ML models, except for the CT-DT ensemble case, where 0.75 was assigned to the CT-based model and 0.25 to the DT model, because this model had binary predictions. The classification threshold (θ) was set to 0.7. As a result, for each couple of models (CT-KNN, CT-LR, CT-DT, and CT-RF), new predictions were generated for each case and, by using the threshold, they were classified as 0 or 1. Confusion matrices and evaluation metrics (accuracy, precision, recall, and F1-score) were also calculated for the ensemble methods.

## 3. Results

Starting with the CT-based model, as presented in Table 2 and Table 3, the model correctly predicts 90% of the cases and also captures all the instances of actual HPV positivity, achieving an accuracy of 90% and recall of 100%, respectively. In the context of HPV diagnosis, it is very important to ensure that positive cases are not missed. In addition, when the model predicts HPV positivity, it is correct 80% of the time, being reliable in positive predictions, and the F1-score of 0.89 suggests a good balance between precision and recall. Overall, the CT-based model appears to perform well, being a useful tool in medical diagnosis concerning HPV status identification. This performance could be attributed to several factors, such as the detailed and rich information provided by the CT data that enables the model to capture complex patterns related to the HPV status. Moreover, as shown in Figure 2, usually, there is no obvious clinical evidence in CT slices, concerning the HPV status, that can be easily observed with the naked eye, and it seems like this model can be used as a supplementary method in this kind of task. In addition, it can be stated that DL architectures, like ResNet-18, are capable of not only learning hierarchical and complex representations by the input data but also automatically learning relevant features directly by the raw CT data. This kind of model appears to be suitable for medical imaging classification tasks.

Regarding the ML models, the logistic regression model shows an accuracy of 60%, indicating a moderate level of overall correctness. Moreover, although the model does not perform very well in predicting the HPV positivity, achieving a precision of 0.57, it can successfully identify 80% of the actual positive cases. However, having a precision equal to 0.57 affects the F1-score, creating an imbalance between precision and recall. Given the fact that it has higher recall than precision, this model seems to be effective at capturing actual positive cases but may have a higher rate of false positives. One reason for this moderate level of performance may be linked to the fact that logistic regression, when dealing with a small dataset, leads to overfitting or challenges in capturing relevant patterns. Also, this model assumes a linear relationship between features and the log-odds of the target variable, so if the relationship is not linear and more complex, it might struggle to represent the underlying complexity.

Regarding the decision tree model, the accuracy and precision are equal to 0.5, meaning that it can correctly predict only half of the cases and half of the positive patients. In addition, recall and F1-score are also low, meaning that the model struggles to make correct predictions. As a result, the model exhibits lower performance, compared to the previous ones. The higher rate of false positives and false negatives indicates that there are challenges in accurately predicting both HPV-positive and HPV-negative patients. Overall, this model does not appear to be appropriate for our task, as it shows poor performance in correctly predicting the cases, with all the metrics being low. The reasons behind this performance vary, with the complexity of the model and the small dataset being possible ones. More specifically, decision trees are susceptible to overfitting, which can result in poor generalization to new, unseen data, leading to low efficacy when applied to the test set.

The random forest model shows 60% accuracy, providing moderate overall correctness. The precision and recall are also 60%, meaning that it has also a moderate ability to predict HPV positivity and successfully identify the actual positive cases. This model exhibits balanced overall performance and appears to provide a good balance between avoiding false positives and capturing positive cases. These results may be influenced by several factors, one of which is the fact that random forest is an ensemble technique that builds numerous decision trees during the training process. The combination of predictions from each tree helps to reduce individual tree biases and overfitting, thus contributing to a more balanced result. This may also be the reason why the random forest model outperforms the decision tree model.

When it comes to the K-Nearest Neighbors model, the accuracy of 80% indicates a relatively high overall correctness. Moreover, the precision and recall are also 80%, meaning that the model is reliable at predicting HPV positivity and successfully capturing the actual positive instances. As a result, the F1-score, which balances these two metrics, is also high (80%). Considering all these metrics, this model demonstrates a balanced and high performance for our classification task, especially when compared to the rest radiomics-based models. One possible reason for the outperformance of the KNN model is the fact that it is a non-parametric model that relies on the local distribution of the data. So, when the patterns in the dataset are to a local approach, where cases of the same class tend to group together, the KNN model has the potential to exhibit high performance. In addition to this, it might also be effective in situations where the relationship in the data is non-linear or more complex. In this case, the other models possibly cannot capture the relevance of the features very well.

As for the models that result from the Weighted Ensemble method, the majority of the new metrics are higher than the metrics of each separately evaluated model. This may be affected by several factors. More specifically, as already mentioned for this method, each model’s prediction is assigned a weight, and the final prediction is a weighted combination of the predictions from all different models. The flexibility to adjust different weights to different models, depending on their performance and reliability, to optimize the ensemble’s performance, provides a powerful tool for fine-tuning. Individual models may have biases or high variances, so this process helps in achieving a more balanced and unbiased final prediction. Taking all the above into consideration, applying a Weighted Ensemble method presents an effective approach to exploit the advantages of each model and limit its weaknesses, achieving better results.

## 4. Discussion

The best classification model was ResNet-18, which was trained with the CTs, demonstrating a promising result of 90% accuracy. On the contrary, the ML models had poor performance with a range of 50 to 60%, except for the KNN model, which showed 80% accuracy. The models that resulted from the Weighted Ensemble method had a moderate level of performance, exhibiting 70 to 90% accuracy.

The performance difference between the CT-based model and the radiomics-based models may be attributed to the different types of data that were used as input. Radiomic techniques involve the application of filters on a predetermined region of interest, usually manually segmented, to generate features. Nonetheless, HPV-positive OPCs are recognized to be linked with areas beyond the tumor volume, such as cystic lymph nodes. Consequently, conventional radiomic methods often overlook crucial aspects of the medical image, whereas DL eliminates the need for delineating the tumor volume, providing an advantage in predicting the HPV status.

Moreover, it is crucial to mention that radiomic features are designed to record precise quantitative attributes related to texture, shape, and intensity in the images. Nevertheless, they may not entirely encompass the complicated information found in CT data, particularly in situations involving pathology or subtle variations, like HPV detection. In addition, feature selection involves reducing the dimensionality of data. Hence, this process could lead to information loss, possibly excluding relevant features that play a role in the comprehensive predictive capability of the model.

In addition, model architecture might play an important role in this study. The structure of a model trained on CT images, particularly if it is a DL architecture such as ResNet-18, might be more adept at acquiring complex representations from high-dimensional data and thus contribute to improved model performance. The small dataset size may also affect the performance of the models, since not only does it provide a limited representation of the general population or the specific patient group of interest, leading to model generalization, but it also increases the risk of overfitting, leading to poor performance on new, unseen data.

Furthermore, it is important to mention that due to the resampling, the CT-based model was trained on over 7000 2D slices, whereas the radiomics-based models were trained on approximately 40 CT data. This probably constitutes an important reason for the radiomics-based models’ poor performance, and perhaps it could have been avoided if the number of patients was larger. However, the application of the Weighted Ensemble method, and thus the combination of the models, results in better accuracy because of the use of the transfer learning approach.

Taking all the above into consideration, it can be stated that the proposed procedure requires further investigation before it could be introduced in the clinical routine as a complementary non-invasive process to predict the HPV status, which can constitute an important diagnostic and prognostic factor for HNC. Nevertheless, this is an early-phase approach that needs more investigation and improvement.

## 5. Conclusions

HNCs present significant challenges for clinicians, because of the complex regional anatomy, frequently small sizes, variability in oncologic pathology, and alterations to the anatomical site post-treatment [23]. This study’s results indicate that radiomics reveal tumor and normal tissue information that is beyond visual analysis, such as the investigated HPV status detection. This usually requires the use of microscopic bioassays that may produce discomfort or stress for the patient. Instead, the proposed models can act as a preliminary test to categorize patients as potential positive/negative HPV cases. However, it is essential to carry out additional validation research using larger and multicenter cohorts to confirm these results and enhance the existing predictive models’ performance. Moreover, research should also concentrate on comparing the 2D approach with various 3D DL methods to evaluate the benefits and the drawbacks of each process. In addition to this, it would be interesting to see the prognostic value of the CT-based model when CT data from healthy patients are included during training. Last but not least, when it comes to the radiomics-based models and the improvement of their accuracy, future work should focus on using more data, including medical images and quantitative features. 

To conclude, if the mentioned challenges can be addressed and studies focus more on the application of radiomics and machine learning for HNCs, there is a possibility of radiomics serving as an economical, non-invasive, and supplementary approach for HPV detection, especially in situations where tissue samples are unavailable or in regions where routine testing is not a common practice.

## Figures and Tables

**Table 1:** Dataset characteristics.

	Training Set			Validation Set	
Clinical Center	CHGJ	CHUP	CHUS	CHUS	CHUP
#Patients	12	12	16	3	7
Age	67 [50–81]	62.3 [46–81]	56.9 [35–77]	65.7 [60–77]	63.8 [49–76]
Gender					
Male	10 (83.3%)	11 (91.7%)	11 (68.8%)	2 (66.7%)	4 (57.2%)
Female	2 (16.6%)	1 (8.3%)	5 (31.2%)	1 (33.3%)	3 (42.8%)
Primary tumor site					
Oropharynx	12 (100%)	12 (100%)	16 (100%)	3 (100%)	7 (100%)
Larynx					
T-stage					
T1/T2	6 (50%)	3 (25%)	9 (56.2%)	2 (66.7%)	2 (28.6%)
T3/T4	6 (50%)	9 (75%)	7 (43.8%)	1 (33.3%)	5 (71.4%)
N-stage					
N0	2 (16.7%)	2 (16.7%)	3 (18.8%)	1 (33.3%)	
N1	3 (25%)	3 (25%)	1 (6.2%)		3 (42.8%)
N2	6 (50%)	7 (58.3%)	11 (68.8%)	2 (66.7%)	2 (28.6%)
N3	1 (8.3%)		1 (6.2%)		2 (28.6%)
M-stage					
M0	12 (100%)	9 (75%)	16 (100%)	3 (100%)	6 (85.7%)
M1		3 (25%)			1 (14.3%)
Overall stage					
Stage I		1 (8.3%)			1 (14.3%)
Stage II		3 (25%)	3 (18.8%)	1 (33.3%)	1 (14.3%)
Stage III	5 (41.7%)	2 (16.7%)			1 (14.3%)
Stage IV	7 (58.3%)	6 (50%)	13 (81.2%)	2 (66.7%)	4 (57.1%)
HPV status					
Negative	6 (50%)	6 (50%)	5 (31.2%)		5 (71.4%)
Positive	6 (50%)	6 (50%)	11 (68.8%)	3 (100%)	2 (28.6%)
Chemotherapy					
No	1 (8.3%)		2 (12.5%)		
Yes	11 (91.7%)	12 (100%)	14 (87.5%)	3 (100%)	7 (100%)

**Table 2:** The predictions of all the models, including the predictions after the WE method, for each case to be 1. Pred: Prediction, WE: Weighted Ensemble, LR: logistic regression, KNN: K-Nearest Neighbors, DT: decision tree, RF: random forest.

Patient ID	Pred CT	CT Label	Pred LR	WE LR	Pred ΚΝΝ	WE KNN	Pred DT	WE DT	Pred RF	WE RF	Target
Test 01	0.06	0	0.84	0.34	1.00	0.40	1	0.30	0.90	0.36	0
Test 02	0.83	1	0.82	0.33	0.80	0.32	0	0.62	0.80	0.32	1
Test 03	0.84	1	0.73	0.29	0.80	0.32	1	0.88	0.70	0.28	1
Test 04	0.99	1	0.73	0.29	0.40	0.16	1	0.99	0.70	0.28	0
Test 05	0.98	1	0.21	0.08	0.40	0.16	0	0.74	0.30	0.12	1
Test 06	0.26	0	0.40	0.16	0.40	0.16	0	0.20	0.10	0.04	0
Test 07	0.40	0	0.51	0.20	0.20	0.08	1	0.55	0.40	0.16	0
Test 08	0.91	1	0.51	0.20	0.60	0.24	0	0.68	0.20	0.08	1
Test 09	0.30	0	0.69	0.28	0.40	0.16	0	0.23	0.50	0.20	0
Test 10	0.14	0	0.42	0.17	0.60	0.24	0	0.11	0.70	0.28	0

**Table 3:** The performance metrics of all the models, as well as their confusion matrix.

Model/Metrics	Accuracy	Precision	Recall	F1-Score	Confusion Matrix [TP, FP], [FN, TN]
CT	0.90	0.80	1.00	0.89	[4, 1], [0, 5]
LR	0.60	0.57	0.80	0.66	[4, 3], [1, 2]
KNN	0.80	0.80	0.80	0.80	[4, 1], [1, 4]
DT	0.50	0.50	0.40	0.44	[2, 2], [3, 3]
RF	0.60	0.60	0.60	0.60	[3, 2], [2, 3]
CT + LR	0.80	0.75	0.75	0.75	[3, 1], [1, 5]
CT + KNN	0.90	0.80	1.00	0.89	[4, 1], [0, 5]
CT + DT	0.70	0.67	0.50	0.57	[2, 1], [2, 5]
CT + RF	0.80	0.75	0.75	0.75	[3, 1], [1, 5]

## Data Availability

The model can be downloaded at https://github.com/EffiePetrou/HPV-status-classification.git (accessed on 7 July 2024). Data can be accessed at https://hecktor.grand-challenge.org (accessed on 7 July 2024).
